# An AI-based pipeline for osteoporosis/osteopenia prediction using hip radiographs

**DOI:** 10.1007/s00256-025-05033-x

**Published:** 2025-09-26

**Authors:** José Acosta-Batlle, David Coronado-Gutiérrez, Javier Soto, Jaime Moujir, Carlos López, Carlos Suevos Ballesteros, Mónica Vázquez Díaz, María del Prado Orduña Díez, David Fernández, Javier Blázquez Sánchez

**Affiliations:** 1 Radiology Department, Hospital Universitario Ramón y Cajal, IRYCIS, Madrid, Spain; 2 Transmural Biotech S.L., Juan Ignacio Luca de Tena 12, 28027 Madrid, Spain; 3 Rheumatology Department, Hospital Universitario Ramón y Cajal, IRYCIS, Madrid, Spain; 4 Nuclear Medicine Department, Hospital Universitario Ramón y Cajal, IRYCIS, Madrid, Spain

**Keywords:** Osteoporosis, Osteopenia, Artificial Intelligence, Deep Learning, Diagnosis, Radiography

## Abstract

**Objective:**

To develop and validate an artificial intelligence-based tool for the diagnosis of osteoporosis/osteopenia using hip radiographs. The tool aims to classify femurs into risk-based categories for osteoporosis/osteopenia, enabling patient prioritization, enhancing preventive medicine through incidental detection, and assisting clinicians’ diagnosis in general.

**Materials and methods:**

The AI tool was designed to perform three preprocessing tasks before the osteoporosis/osteopenia prediction: (1) splitting images into single femurs, (2) identifying and discarding femurs with prostheses, and (3) cropping images to isolate the proximal femur. A total of 2691 anteroposterior hip radiographs from 1654 patients were included in the study. The osteoporosis/osteopenia prediction model was trained on 3227 single femur images and tested on 826. Additionally, a final evaluation experiment was conducted on 313 new radiographs from 239 patients to assess the tool’s applicability.

**Results:**

The tool demonstrated high performance in the preprocessing tasks, achieving 99.0% accuracy in classifying single vs. double femur images, 99.3% accuracy in identifying prosthetic femurs, and 99.2% pixel accuracy in delineating the proximal femur before cropping. The final prediction model achieved an area under the curve of 86.6% for detecting osteoporosis/osteopenia in the test set and 81.0% in the final evaluation experiment.

**Conclusions:**

The obtained results demonstrate the potential of the proposed AI-based pipeline for prediction of osteoporosis/osteopenia using hip radiographs. This study suggests that a tool based on the proposed methods could support DXA triage, incidental osteoporosis detection, and clinical decision-making in settings with limited access to bone densitometry.

## Introduction

Osteoporosis is a chronic metabolic bone disease characterized by decreased bone mineral density (BMD) and increased bone fragility, leading to a higher risk of fractures. Osteoporosis is often asymptomatic until a fracture occurs, making early diagnosis essential [1]. The global prevalence is 18.3%, higher in women (23.1%) than in men (11.7%), and rises to 35.3% in the elderly [2, 3]. Fragility hip fractures—one of its most serious consequences—occur in 100–200 per 100,000 individuals annually, often leading to long-term disability and increased mortality [4–7].

Despite this, osteoporosis is preventable and manageable through early diagnosis and lifestyle modifications [1]. Notably, nearly half of fractures in individuals over 55 occur in those with osteopenia, the precursor to osteoporosis, highlighting the need for early detection [8]. Dual-energy X-ray absorptiometry (DXA) is the gold standard for diagnosis, based on T-scores below − 1 (osteopenia) and − 2.5 (osteoporosis) [9, 10]. While DXA has proven effective in reducing fracture and mortality rates [11–13], its limited availability, cost, and low awareness restrict its widespread use [11, 14].

Conventional radiographs, which are more accessible and frequently performed, hold promise for osteoporosis/osteopenia detection, particularly when enhanced by artificial intelligence (AI). Integrating radiographs into screening strategies could enhance efficiency and broaden access to diagnostic tools, particularly in resource-limited settings [15]. Several studies have demonstrated the potential of AI models to detect osteoporosis from conventional hip radiographs, achieving accuracy rates ranging from 62.3 to 98.1% and areas under the curve (AUC) values of up to 97.0% [16–21]. However, these models typically focus only on osteoporosis, overlooking osteopenia—a key stage for early intervention—, as if it has been explored with other types of radiographs [22–25]. Furthermore, among the cited studies, only CI. Hsieh et al. [17] presents a fully end-to-end automated pipeline suitable for integration into routine clinical workflows.

Therefore, this study aims to develop and validate an automated AI-based pipeline for detecting both osteoporosis and osteopenia using hip radiographs, designed to be robust and adaptable to real-world clinical conditions.

## Materials and methods

The institutional review board of Hospital Universitario Ramón y Cajal in Madrid (Spain) approved this study (code 329–21). Due to the study’s retrospective design, the institutional review board waived the need for informed patient consent.

### Data collection

A retrospective collection of hip radiographs and corresponding BMD measurements taken from November 2016 to June 2023 (global study) and from June 2023 to April 2024 (final evaluation set) were conducted at Hospital Universitario Ramón y Cajal.

The dataset included standard anteroposterior hip radiographs of one or both sides of the pelvis. Radiographs in other projections (such as axial, lateral, oblique, or posteroanterior views) and low-quality images (poor acquisition parameters, digital artifacts, degraded digitized printed plates, excessive noise, incomplete visualization of the region of interest (ROI), or object superposition) were excluded by a clinical expert. Notably, images with other pathologies (apart from osteoporosis/osteopenia) have also been included in order to obtain a more complete tool. Images with prosthesis (or other metal implants) were used to train an algorithm to discard them in the final tool, but they were not used in the final prediction algorithm.

**Fig. 1:**
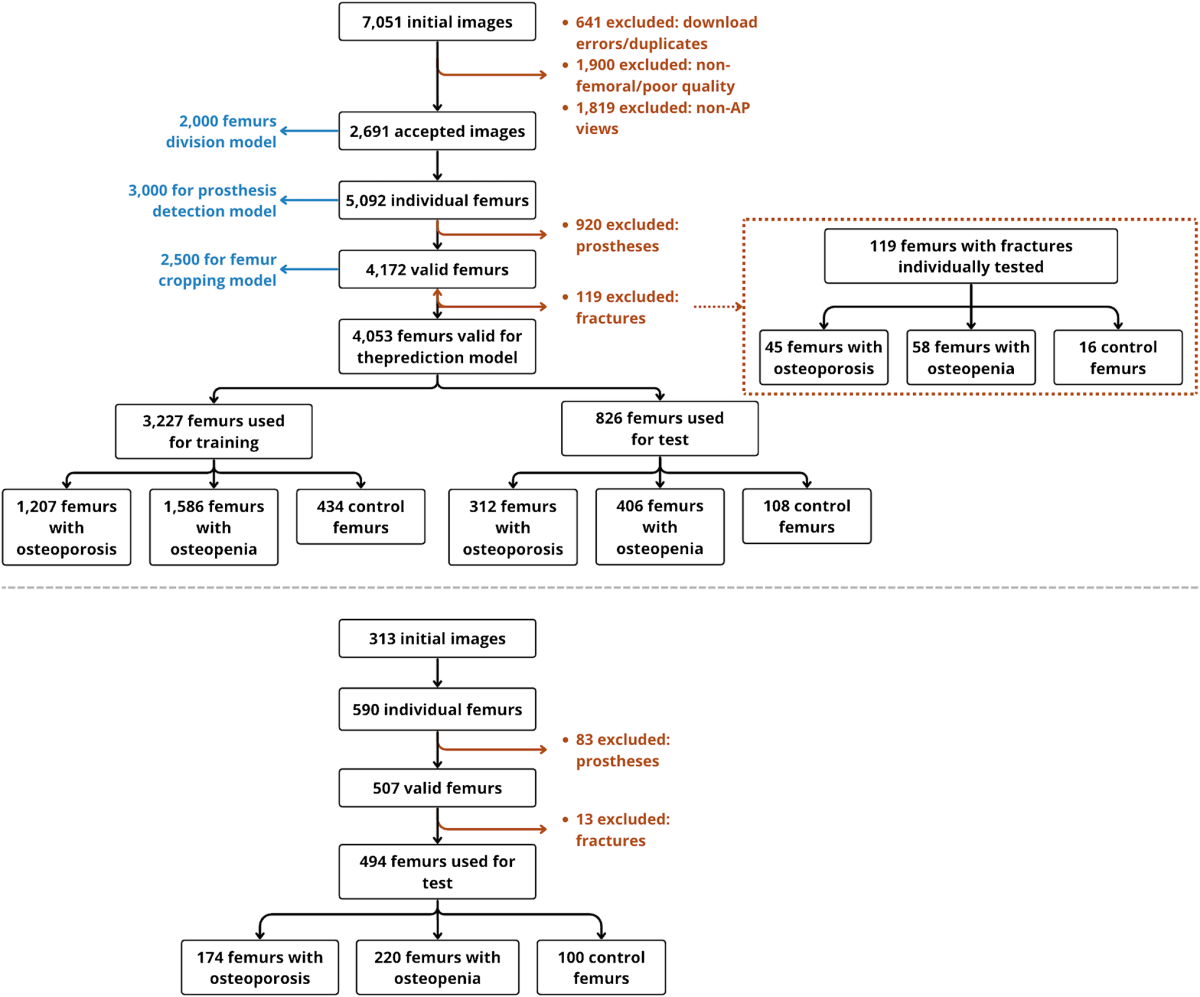
Flowchart illustrating the dataset processing pipeline, from initial image collection to final dataset distribution for training and testing (top side) and the final evaluation (bottom side). Exclusion criteria and preprocessing steps for osteoporosis/osteopenia prediction are included

In total, 7051 radiographs were initially collected, of which 2691 images from 1654 patients were finally included in the global study for model development and internal testing (Fig. 1, top side). For the final evaluation, an independent set of 313 validated images from 239 patients was used, after excluding images with other views or insufficient quality (Fig. 1, bottom side). This final evaluation set was employed to assess the fully developed system under conditions simulating real-world deployment and also to compare them with the prediction that clinicians would make by naked eye. Demographic data were collected for each patient of both sets (Table 1).

**Table 1:** Patient characteristics. Mean data are expressed with standard deviations. *P* values are computed with Mann–Whitney *U* test [26] (age, weight, and days) and chi-squared test [27] (sex and BMD). (*BMD*, bone mineral density)

Characteristic	Global study set	Final evaluation set	*P* value
No. of patients (no. of images)	1654 (2691)	239 (313)	
Age (years)	72.9 ± 10.6	72.3 ± 11.3	0.81
Sex			< 0.052
Women	1499 (90.6%)	207 (86.6%)	
Men	155 (9.4%)	32 (13.4%)	
Weight (kg.)	66.3 ± 16.6	66.3 ± 15.2	0.82
Days between DXA and X-ray	138 ± 204.9	396.5 ± 98.5	0.98
BMD categories			0.216
Normal	203 (12.3%)	39 (16.3%)	
Osteopenia	872 (52.7%)	120 (50.2%)	
Osteoporosis	579 (35.0%)	80 (33.5%)	

### Imaging protocol

Radiographs were acquired using various digital radiography systems, including Optimus 50 (Philips, Amsterdam, Netherlands), Medio 50 CP-H (Philips, Amsterdam, Netherlands), DigitalDiagnost C90 (Philips, Amsterdam, Netherlands), I-RAD/NET 400 (Toshiba, Tokio, Japan), Indico 100 RAD (Toshiba, Tokio, Japan), SHF535 (Toshiba, Tokio, Japan), FDR D-EVO GL (Fujifilm, Tokio, Japan), and Ysio Max (Siemens, Berlin, Germany). Radiologic technologists followed established clinical guidelines to obtain all radiographs using peak kilovoltage (kVp) values ranging from 50 to 125.

### Bone mineral density assessment

BMD measurements, along with bone mineral content (BMC), T-score, and Z-score, were obtained using DXA at the same institution. The DXA equipment included Hologic Horizon WI and Hologic Explorer densitometers (Hologic Inc., Marlborough, MA, USA). The DXA referred specifically to hip densitometry, and the parameters of BMC, T-score, and Z-score were measured at the femoral neck. These measurements were performed within a 2-year interval relative to the date of the corresponding radiograph [28, 29].

The radiographs were classified based on T-score in accordance with the World Health Organization criteria for diagnosing osteoporosis [30]: normal (T-score higher or equal to − 1.0), osteopenia (T-score lower than − 1.0 and higher than − 2.5), and osteoporosis (T-score lower or equal to − 2.5). In instances where radiographs included both femurs, but T-score was available for only one femur, the same classification was applied to both femurs [31].

### Development of AI model

#### Processing pipeline

**Fig. 2:**
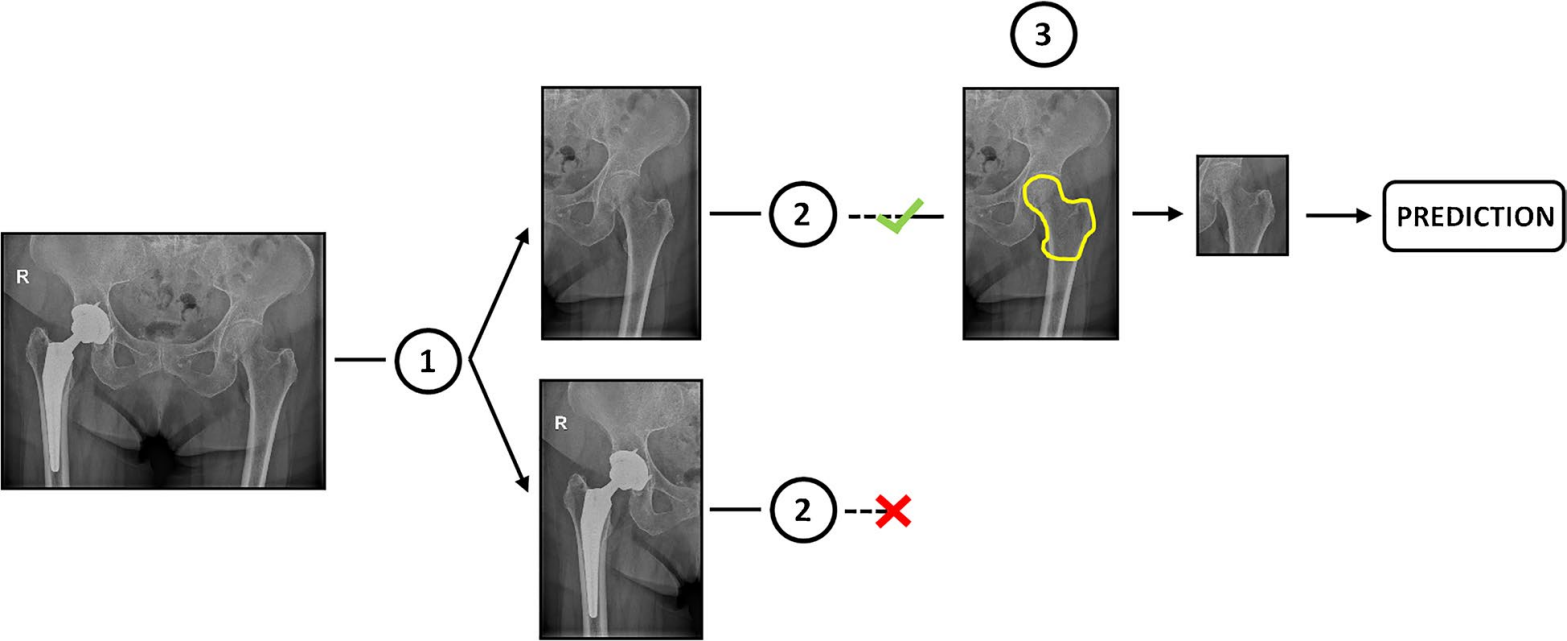
Complete pipeline of the study composed of the final osteoporosis/osteopenia prediction and the three previous preprocessing steps: (1) split the images in single femurs; (2) discard femurs with prosthesis; and (3) crop the images by the proximal extremity of the femur

This study aimed to develop a deep learning-based tool to predict osteoporosis/osteopenia in hip X-rays, using a four-step pipeline that includes three preprocessing stages (split the images in single femurs, discard femurs with prosthesis, and crop the images by the proximal extremity of the femur) and the final disease prediction (Fig. 2).

It is important to note that this section only describes the deep learning models used in the final tool. A wide range of models —including both segmentation and classification approaches—were initially explored, and those yielding the best performance were finally selected.

#### Division into two femurs

To obtain single-femur images, a deep learning model was trained to classify radiographs as showing one or both sides of the pelvis. Images with both sides were automatically split vertically into two separate images (right and left femur) of equal size, while those with only one femur were left unchanged. This process converted the entire dataset into standardized single-femur images.

To perform this classification task, a ResNet-18 (Residual Neural Network) [32] was trained with a set of images labeled as “one side” or “two sides” by technicians. Once trained, the resulting model was tested on another set of labeled images to see its classification performance. ResNets are CNNs pre-trained with ImageNet dataset [33]. For this purpose, the network was trained during ten complete passes through the dataset (epochs) with batches of 32 samples (batch size). Furthermore, softmax loss function [34], Adam optimization algorithm [35], and batch normalization [36] were also used.

#### Prosthesis detection

To prevent interference in the osteoporosis/osteopenia prediction, a deep learning model was trained to automatically detect and discard femurs with prostheses by classifying each image as “prosthesis” or “non-prosthesis.”

To perform this classification task, another ResNet-18 [32] was trained with a set of images (single femurs) labeled as “prosthesis” or “non-prosthesis” by technicians. Once trained, the resulting model was tested on another set of labeled images to see its classification performance. For this purpose, the network was trained with the same configuration as the previous one: ten complete passes through the dataset, batches of 32 samples, softmax loss function [34], Adam optimization algorithm [35], and batch normalization [36].

#### Femur cropping

To focus on the proximal extremity of the femur—where disease-related features are most evident—a deep learning model was trained to segment this region. Based on the segmentation, each femur was automatically cropped using a rectangular area that encompassed the delineated region and its surroundings (Fig. 2, step 3).

To perform this delineation task, a DeepLab [37] CNN was trained with a set of delineations. Three specialists previously delineated manually the proximal extremity of some femur with an online graphical user interface created by Transmural Biotech (Madrid, Spain) [38]. Once trained, the learning model was tested on another set of delineated images to see its segmentation performance. DeepLab CNN uses an ImageNet [33] pre-trained VGG [39] as its main feature extractor network. Data augmentation was also used to increase the amount of data to train the model and get better results, modifying the contrast, brightness, and saturation of the original images and also mirroring the images horizontally. The CNN was trained during 40 complete passes through the dataset (epochs) with batches of 64 samples (batch size), and softmax loss function [34] and gradient descent optimization algorithm [40] were also used.

#### Osteoporosis/Osteopenia prediction

To finally predict osteoporosis/osteopenia, another deep learning algorithm was trained and tested with all the cropped images together with their corresponding femur outcome (normal, osteopenia, or osteoporosis). Femurs with fractures were manually selected and discarded from the train and test sets to avoid confusing the algorithm, as such cases are known to distort BMD estimation and are typically not suitable for clinical densitometry assessment [41]. Once the model will be trained, these particular images will also be tested on an independent set to observe its performance.

To account for variability in acquisition conditions, all cropped images were previously normalized using contrast limited adaptive histogram equalization (CLAHE) to standardize gray levels and enhance image contrast [42].

Finally, to perform this osteoporosis/osteopenia prediction task, a DenseNet-169 CNN [43] was trained with 80% of the final dataset and tested on the remaining 20% to see its classification performance. No patient contributed images to both sets to prevent data leakage. For this purpose, the network was trained during 40 complete passes through the dataset (epochs) with batches of 32 samples (batch size). Furthermore, softmax loss function [34], Adam optimization algorithm [35], and batch normalization [36] were also used.

#### Statistical analysis

The performance of each of the steps was evaluated by comparing results of each experiment with the manual ground-truth on the test set images. Statistical analysis was performed using MATLAB R2023a.

To evaluate the two first experiments (“division into two femurs” and “prosthesis detection”), the accuracy (percentage of correct predictions) was computed. To assess the “femur cropping” experiment, pixel accuracy (percent of total pixels correctly classified) and Dice coefficient [44] were computed to compare the obtained automated ROIs with the manual ROIs made by our clinicians. Finally, to evaluate the osteoporosis/osteopenia prediction experiment, the receiver operating characteristic (ROC) curve and the typical classification metrics were computed: accuracy, sensitivity, specificity, positive predictive value (PPV), and negative predictive value (NPV).

To avoid providing a binary diagnosis and to increase the interpretability and flexibility of the model’s output, a 5-class prediction system is proposed: class 1 corresponds to very high sensitivity (> 99%); class 2 to high sensitivity (> 80%); class 3 an intermediate balance between sensitivity and specificity; class 4 to high specificity (> 95%); and class 5 to very high specificity (> 99%). This classification allows clinicians to interpret results in the context of their specific clinical priorities. For example, if the goal is to detect as many potential osteoporosis cases as possible (minimizing false negatives), then actions may be taken based on class 1 alone or classes 1 and 2. In contrast, if the aim is to minimize false positives, only classes 4 or 5 may be considered. The thresholds for these classes were determined by evaluating the PPV and NPV in a simulated population with a prevalence matching the global prevalence of osteopenia and osteoporosis. Thresholds were selected considering the number of hip radiographs and DXA performed at our center, ensuring practicality and relevance from a cost-efficiency perspective.

## Results

### Preprocessing experiments

In the first experiment, the proposed classification learning method was used to classify the incoming images as “one femur” or “two femurs” images. One thousand eight hundred images were used to train the deep learning algorithm, and 200 images (122 with two femurs and 78 with a single femur) were used to test its performance. The final model correctly classified 198 of the 200 images (99.0% of accuracy), misclassifying two images of single femur as two-femur images.

In the second experiment, the proposed classification learning method was used to classify the incoming images (single femurs) as “prosthesis” or “non-prosthesis”. Two thousand seven hundred images were used to train the deep learning algorithm, and 300 images (38 femurs with prosthesis and 262 without prosthesis) were used to test its performance. The final model correctly classified 298 of the 300 femur images (99.3% accuracy), failing to detect two femurs with prostheses.

In the third experiment, the proposed segmentation learning method was used to automatically delineate the proximal extremity of the femur from the incoming images (single femurs without any prosthesis). Two thousand two hundred fifty images were used to train the deep learning algorithm, and 250 images were used to test its performance. The final model achieved a pixel accuracy of 99.2%, with a Dice coefficient of 95.0%.

### Osteoporosis/Osteopenia prediction

In the final experiment, the proposed classification learning method was used to predict osteoporosis/osteopenia disease from the incoming images (cropped proximal extremity of the femur). A total of 4172 images were used: 1563 with osteoporosis, 2051 with osteopenia, and 558 without any of these diseases (controls). From the total, 3227 images (77%) were used to train the deep learning algorithm and 826 images (20%) to test its performance, maintaining the prevalence of each class. Finally, 119 femurs with fractures (3%) were also tested independently to see the performance over these special cases. Figure 1 (top side) shows the dataset distribution for this final experiment.

**Fig. 3:**
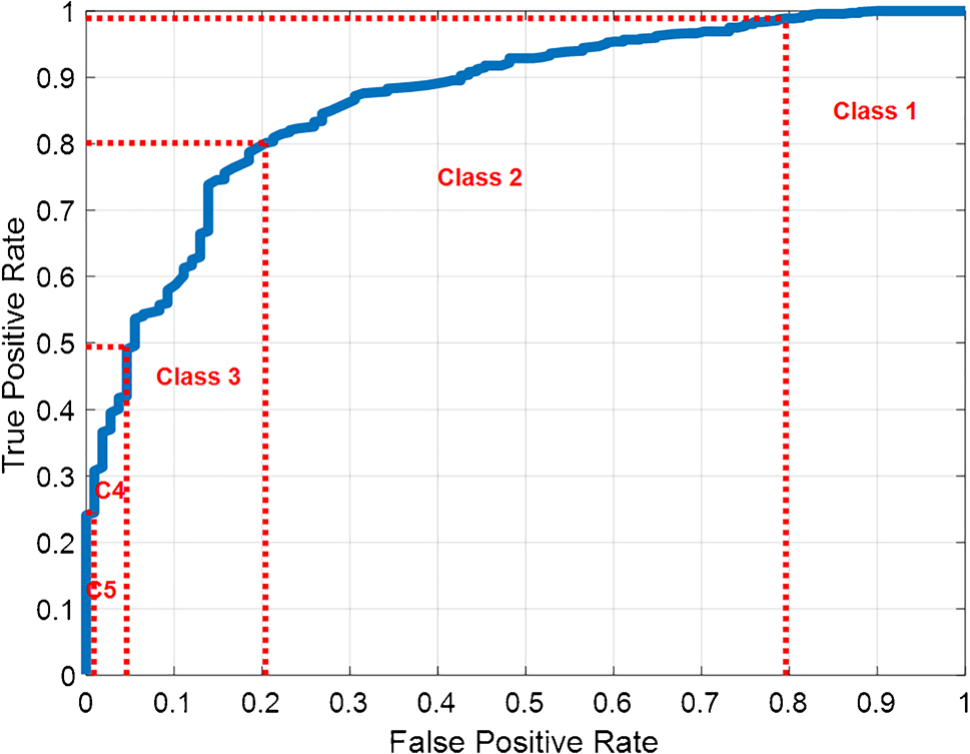
ROC curve obtained by the proposed method for prediction of osteoporosis/osteopenia on the test images with the division of the five proposed classes

Figure 3 shows the ROC curve obtained by the proposed classification learning method which achieved an AUC of 86.6%. Figure 3 also shows the division of the curve into the five proposed classes. Table 2 shows the typical classification metrics of each cutoff point of the ROC curve.

**Table 2:** Classification metrics of the proposed method for prediction of osteoporosis/osteopenia at each cutoff point of the ROC curve of Fig. 3. (*ACC*, accuracy; *SENS*, sensitivity; *SPEC*, specificity; *PPV*, positive predictive value; *NPV*, negative predictive value)

Cutoff point	ACC	SENS	SPEC	PPV	NPV
Classes 4–5	34.3 (283/826)	24.5 (176/718)	99.1 (107/108)	99.4 (176/177)	16.5 (107/649)
Classes 3–4	55.5 (458/826)	49.4 (355/718)	95.4 (103/108)	98.6 (355/360)	22.1 (103/466)
Classes 2–3	80.0 (661/826)	80.1 (575/718)	79.6 (86/108)	96.3 (575/597)	37.6 (86/229)
Classes 1–2	88.6 (732/826)	98.9 (710/718)	20.4 (22/108)	89.2 (710/796)	73.3 (22/30)

**Fig. 4:**
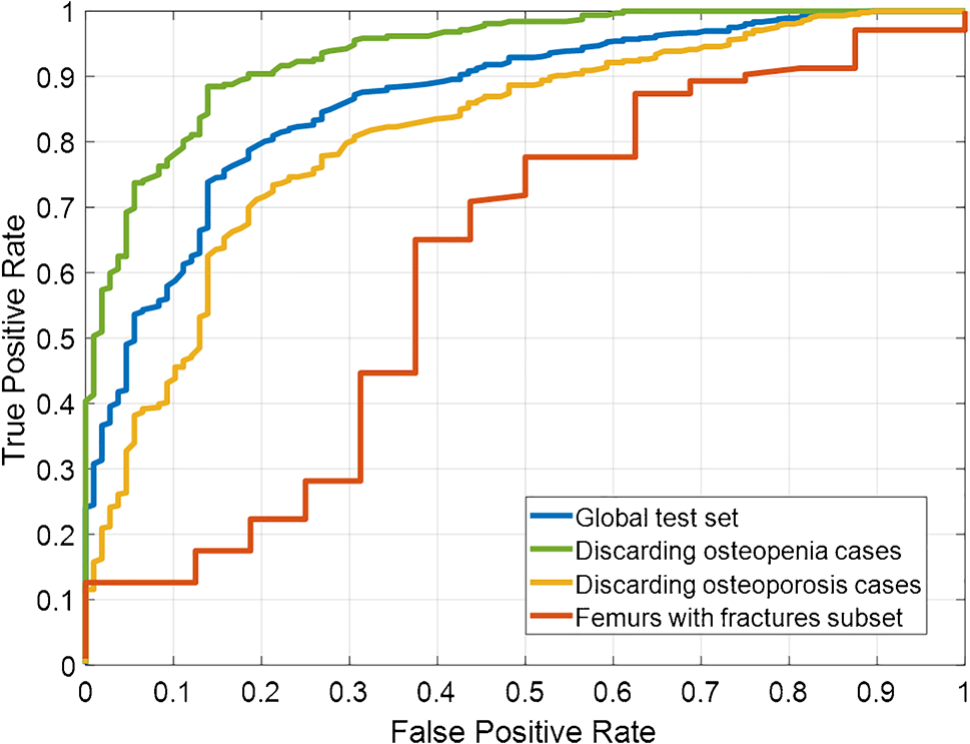
ROC curves obtained by the proposed method for prediction of osteoporosis/osteopenia. Blue line, global test set; green line, test set discarding osteopenia cases; yellow line, test set discarding osteoporosis cases; red line, femurs with fractures subset

To observe the differences in results obtained when predicting cases with osteopenia and cases with osteoporosis, Fig. 4 shows two different ROC curves: green line shows the ROC curve obtained discarding osteopenia cases (the overall AUC rose to 93.6%) and yellow line the curve obtained discarding osteoporosis cases (the AUC fell to 81.2%). Figure 4 also shows the ROC curve obtained testing only the femurs with fractures which were isolated from the global test set (red line). In this case, the AUC achieved was 61.4%.

### Final evaluation results

Once the results of each of the main tasks of our tool have been obtained, a final algorithm is built with the four AI models created (one for each task). This algorithm will automatically apply the four models one after the other, to obtain a final prediction for each new image. The results of the final evaluation of this algorithm for a set of 313 new images (acquired after the previous ones) from 239 patients are shown below.

Of the total set of 313 images, 277 were images containing both sides of the pelvis and 36 were images with a single femur. The algorithm automatically classified these images in one or two femurs, achieving an accuracy of 100% (all 313 were correctly classified). Then, the algorithm divided the images of two femurs into two different images, resulting in 590 different images of single femurs.

Of these 590 resulting femurs, 87 had prosthesis. The algorithm automatically classified these femur images in “prosthesis” or “non-prosthesis,” achieving an accuracy of 99.3% (four femurs with some metal implants were not detected). Then, the algorithm discarded the femur images with prosthesis detected, resulting in 494 images.

Subsequently, the algorithm automatically segmented the ROI and cropped the proximal extremity of the femur. Of the 494 femur images, the algorithm correctly cropped 498 images (98.2%); in eight images (1.6%), part of the proximal extremity of the femur was cut off, and one image (0.2%) had a totally incorrect cropping.

**Fig. 5:**
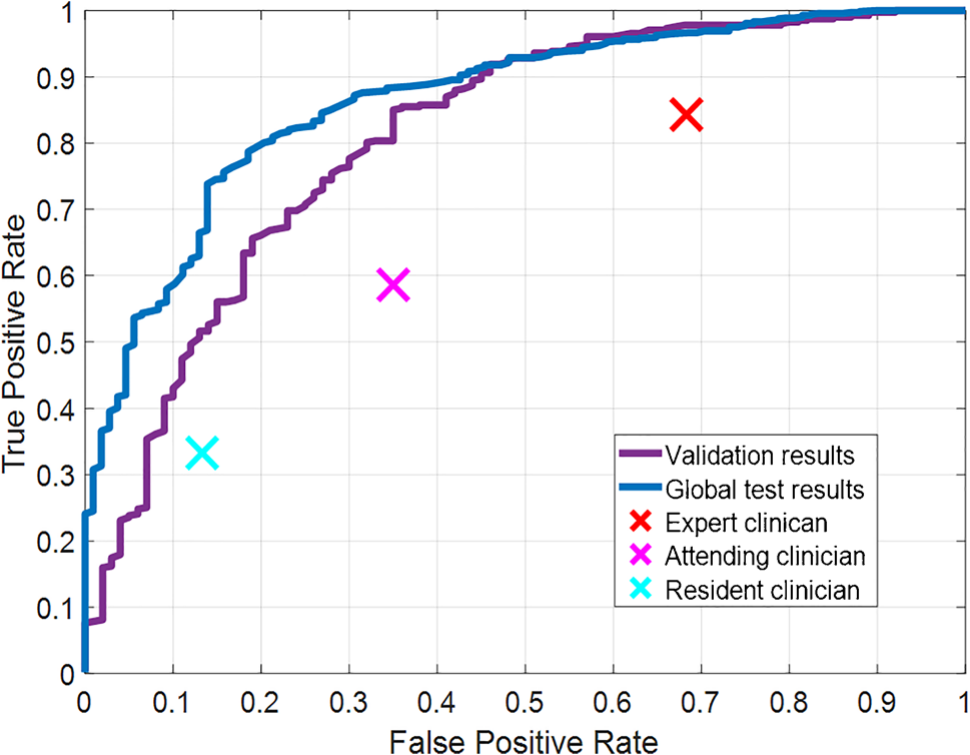
ROC curves obtained by the proposed method for prediction of osteoporosis/osteopenia on the final evaluation set images (purple line) and the global test images (blue line). The crosses indicate the results obtained by the expert clinician (red), attending clinician (magenta), and resident clinician (cyan)

Finally, the algorithm predicted osteoporosis/osteopenia disease from the resulting images (without taking into account the 13 fractured femurs). Of the 494 cropped images, 174 were images from femurs with osteoporosis, 220 from femurs with osteopenia and 100 femurs without any of these diseases (controls). Figure 5 shows the ROC curve obtained by the proposed algorithm on the final evaluation set, which reached an AUC of 81.0%. To compare the differences with the global results, Fig. 5 also shows the previous ROC curve obtained from the global test set (AUC of 86.6%).

To further validate these results, three clinicians with varying levels of experience were asked to provide a visual diagnosis of osteopenia/osteoporosis based solely on the radiographs of the 311 images. The resulting sensitivity/specificity values per femur were 84.3%/31.7% (expert clinician, 24 years of experience), 58.6%/65.0% (attending clinician, 2 years of experience), and 33.3%/86.7% (second year resident clinician). Figure 5 includes crosses representing these results for comparison with those obtained by the algorithm.

## Discussion

This study presents an AI-based tool for osteoporosis/osteopenia prediction from hip radiographs, consisting of four automated steps: femur division, prosthesis filtering, proximal femur cropping, and disease classification.

The results obtained in the three preprocessing experiments are outstanding, as evidenced by the high-performance metrics achieved. The classification of input images in single or two femurs and the classification of femurs in prosthesis or non-prosthesis both achieved an accuracy of 99%, demonstrating that the applied learning methods are effective to perform automatically the tasks of splitting images in femurs and discarding prosthetic femurs. The observed errors were primarily associated with the presence of small intramedullary nails in prosthetic cases, and with atypically rotated images affecting femur count estimation. Further improving the model’s robustness should involve augmenting the training dataset including a greater variety of such edge cases. Additionally, the automatic delineation of the proximal extremity of the femur obtained an accuracy of 99.2% and a Dice coefficient of 95.0%, indicating that the segmentation methods used are efficient enough to automatically delineate this femur region, ensuring a high-quality crop of the ROI.

Analyzing the main results for osteoporosis/osteopenia prediction, the ROC curve obtained with an AUC of 86.6% shows promising potential. Comparisons with similar studies are difficult, as most existing work focuses on predicting osteoporosis risk alone, rather than predicting both osteoporosis/osteopenia simultaneously. Among studies on dual osteoporosis/osteopenia detection [22–25], they reported AUC between 72.6 and 91.0%, using other types of radiographs (lumbar spine, chest, and knees), marginally better than our results. Other studies report AUCs for only osteoporosis predictions that are in a comparable range to our study (between 70 and 97% [16–21]). More specifically, the study of S. Kim et al. [18] achieved an AUC of 96% with a sensitivity and specificity of 82% and 89% respectively, on an internal set of 457 radiographs. With a similar specificity (90.7%), the method proposed in our study achieved only a 57.9% of sensitivity, to predict osteoporosis/osteopenia on an internal set of 826 femurs.

Subgroup analysis shows that excluding osteopenia cases improves AUC, while excluding osteoporosis cases reduces performance, indicating the model is more effective at detecting osteoporosis. This aligns with expectations, as osteoporotic femurs are more radiographically distinguishable from healthy ones, whereas osteopenia represents a subtler, intermediate stage, difficult to detect in X-ray images until a substantial amount of bone loss has already occurred [45]. Furthermore, the mild demineralization seen in osteopenia often overlaps radiographically with normal bone, both visually and algorithmically [46]. These subtleties limit the ability of AI models to reliably detect osteopenic changes, as also suggested in recent deep learning studies focused on hip radiographs [16].

Additionally, in the test carried out only on femurs with fractures, it can be observed that the results obtained are significantly worse, indicating that fractured femurs —whether classified as positive or negative for the disease—may introduce confusion to the system. Therefore, it seems reasonable to consider excluding this type of femurs in the final tool to enhance prediction reliability.

Finally, an analysis of the results obtained on the final evaluation set reveals consistency with those on the global test set. Classification accuracy for identifying one or two femurs and prosthesis femurs reached 100% and 99.3% respectively, and 98% of the ROI delineations were correctly completed. Additionally, the osteoporosis/osteopenia prediction showed a ROC curve comparable to the global test, with an AUC reduction of 6.3%. Moreover, in this final evaluation stage—which mirrors the way the tool would be used in real clinical practice—errors can propagate from one task to the next, potentially affecting the final osteoporosis classification. To mitigate this, the implemented tool generates a report that includes the femur division, prosthesis exclusion (if applicable), and the segmentation mask. This transparent reporting allows clinicians to visually inspect the intermediate results and identify any anomalies that might compromise the reliability of the final prediction (such as fracture inclusion). Lastly, the algorithm outperforms all three clinicians’ visual inspection across the entire ROC curve. Variability among clinicians is expected due to differences in experience and the inherent limitations of radiographic diagnosis, particularly given the high prevalence of osteopenia, which complicates visual assessment.

From a clinical standpoint, this study supports the idea that a tool with the proposed AI methods to predict osteoporosis/osteopenia could be highly useful in clinical practice. Categorizing results into five distinct risk classes enhances the tool’s adaptability across diverse clinical uses, offering a level of stratification not commonly explored in other AI-based models for osteoporosis detection. This flexibility helps mitigate the impact of certain misclassifications, while reinforcing that the tool is intended solely as a clinical decision support system —not as a replacement for clinical judgment or a standalone diagnostic solution—. One of the primary applications is triage for DXA prioritization, particularly in healthcare settings with limited access to bone densitometry. In this approach, patients would be prioritized from the highest risk (class 5) to the lowest risk (class 1). This selective approach could improve the efficiency of osteoporosis screening in settings where routine DXA for all at-risk patients is not feasible. A second potential application is incidental detection, where osteoporosis/osteopenia is identified in patients undergoing hip radiography for unrelated indications. In this setting, patients classified in higher-risk classes would be strong candidates for confirmatory DXA testing to determine the need for pharmacological treatment. Each center may define which categories to consider high risk, depending on cost and DXA availability. Finally, the tool could assist clinicians in their diagnosis in general, for example when other diagnostic methods, such as DXA, are inaccessible. Clinicians could define a risk threshold (class) that best aligns with local healthcare priorities, optimizing sensitivity or specificity based on the patient population and available treatment resources.

This study presents several strengths. First of all, all images were acquired during routine clinical practice by different operators using various machines, with no specific instructions. This approach closely simulates the future clinical scenario that the final tool will likely encounter. Additionally, a final evaluation experiment has also been carried out to observe how the final tool’s performance would evolve across successive tasks. Secondly, unlike most studies, this work aims to predict osteopenia in addition to osteoporosis, allowing an early-stage prediction of the pathology. Finally, all components of the proposed system are fully automated. However, the current implementation still relies on manual exclusion of cases with hip fractures or low-quality images (either before or after reviewing the final report). Despite this limitation, the system is designed in a way that facilitates future integration into clinical software, with the goal of ultimately requiring the operator solely to perform image acquisition.

The main limitation of the study lies in the single-center design and the robustness of the acquired data. Although X-rays from clinical practice were used with different machines, a multicenter study encompassing diverse populations was not conducted. The final evaluation experiment was not tested either on an external database or with prospective data, which reduces the reliability of replicating the results in other clinical environments. Future work should aim to perform external validation through a multicenter study.

In conclusion, the obtained results demonstrate the potential of the proposed AI methods for the prediction of osteoporosis/osteopenia using hip radiographs. This study suggests that a tool with the proposed methods could improve the clinical practice in different clinical uses or scenarios. However, further multicenter studies are necessary to confirm the tool’s reliability and applicability in diverse clinical settings.

## Data Availability

Due to confidentiality agreements, the data supporting this study are not available for public access.

## Abbreviations

DXA: Dual-energy X-ray absorptiometry
BMD: Bone mineral density
CT: Computer tomography
MRI: Magnetic resonance imaging
AI: Artificial intelligence
CNN: Convolutional neural network
AUC: Area under the ROC curve
BMC: Bone mineral content
ResNet: Residual neural network
ROI: Region of interest
ROC: Receiver operating characteristic
PPV: Positive predictive value
NPV: Negative predictive value
